# Controllable Reconstruction of β-Bi_2_O_3_/Bi_2_O_2_CO_3_ Composite for Highly Efficient and Durable Electrochemical CO_2_ Conversion

**DOI:** 10.1021/acs.nanolett.5c00417

**Published:** 2025-04-09

**Authors:** Yuxuan Xiao, Di Liu, Jiao Yang, Jinxian Feng, Wenhao Gu, Lulu Qiao, Weng Fai Ip, Hui Pan

**Affiliations:** † Institute of Applied Physics and Materials Engineering, University of Macau, Macao, SAR 999078, China; ‡ Department of Physics and Chemistry, Faculty of Science and Technology, University of Macau, Macao, SAR 999078, China

**Keywords:** bismuth, controllable reconstruction, directional electron transfer, electrochemical CO_2_ reduction reaction

## Abstract

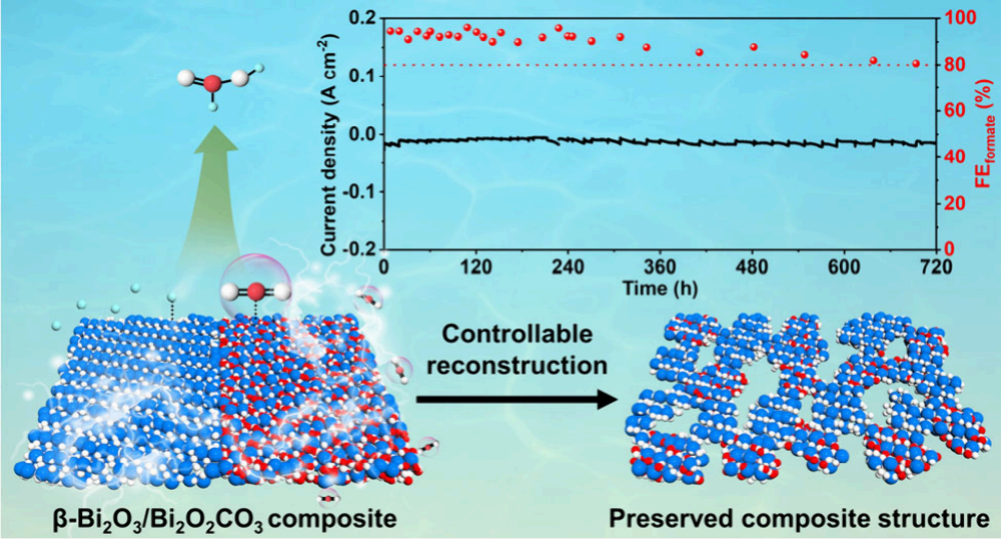

The uncontrollable electrochemical reduction reconstruction, leading to the destruction of well-defined structure and subsequent low durability, is the main obstacle to the catalytic performance of Bi-based composites toward electrochemical CO_2_ reduction reaction (eCO_2_RR). Herein, we address this issue through construction of a novel β-Bi_2_O_3_/Bi_2_O_2_CO_3_ composite, which can resist the reduction reconstruction of Bi-based materials to metallic Bi during the eCO_2_RR process by modulating a more alkaline microenvironment that facilitates the formation of new Bi–O bonds. The synergistic interactions and directional electron transfer between the β-Bi_2_O_3_ and Bi_2_O_2_CO_3_ components, together with the stable composite structure, result in its superior activity and selectivity for formate production with high faradaic efficiencies (FEs) over 94% from −0.7 to −1.1 V, and remarkable durability with maintenance of 80% FE after continuous electrocatalysis of 720 h. This work sheds new light on designing advanced high-performance nanomaterials toward eCO_2_RR and other practical applications.

In the light of the challenges of global warming and intensive energy demands, electrochemical CO_2_ reduction reaction (eCO_2_RR) has emerged as a promising method to convert CO_2_ into high-value-added chemicals, such as CO, methane, methanol, formic acid, and C_2_ products.^1−3^ Among these, formic acid is of particular interest due to its wide range of industrial applications and potential as an energy carrier for fuel cells.^4−6^ Various metal-based electrocatalysts, including Bi,^7−9^ Sn,^10,11^ In,^12−14^ Cu,^15−17^ Sb,^18^ and Pd,^19^ have been investigated for their potential applications in eCO_2_RR, where Bi-based nanomaterials are highly promising due to their high efficiency for formic acid (or formate) production, nontoxicity, and cost-effectiveness.^20^ However, further enhancing the eCO_2_RR activity of Bi-based catalysts for practical applications remains a significant challenge, given issues such as weak CO_2_ adsorption, low electrical conductivity, and slow mass transfer of CO_2_ molecules.^21^ Moreover, Bi-based catalysts can also promote hydrogen evolution reaction (HER) with the same working conditions of eCO_2_RR due to their high reactivity toward proton absorption (*H), leading to a challenge for high selectivity of eCO_2_RR.^22^

To address these challenges, construction of Bi-based composites has garnered significant attention in the field of eCO_2_RR.^23^ By integrating different components, many efforts aim to leverage synergistic active sites and facilitate directional electron transfer to optimize the adsorption/desorption behaviors toward reactants and key intermediates, improving activity, selectivity, and electrical conductivity of materials.^24,25^ Hence, various Bi-based composites, such as Janus structures,^26,27^ core/shell structures,^28−30^ and multi-interface structures,^31−33^ have been developed to achieve improved eCO_2_RR performance. However, the stability of Bi-based composites remains a concern, because Bi-based compounds such as bismuth oxide,^34^ bismuth sulfide,^35^ bismuth oxycarbonate (Bi_2_O_2_CO_3_, BOC),^36^ and bismuth oxyhalide^37^ are vulnerable to reconstruction and/or reduction at high voltages during the eCO_2_RR process, leading to changes of compositions and structures, possible destruction of well-built composites, and thus poor electrochemical durability.^38^ Although the durability over 100 h for Bi-based compounds in eCO_2_RR had been reported in a few works,^39−41^ how to maintain a durable structure has critical implications for the rational design of catalysts with consistent and long-lasting catalytic performance.

Herein, the controllable reconstruction of Bi-based nanomaterials is presented for eCO_2_RR. Initially, a novel β-Bi_2_O_3_/Bi_2_O_2_CO_3_ nanoflower (BO/BOC) with assembled nanosheet composite structure was designed and synthesized. The directional electron transfer from β-Bi_2_O_3_ to Bi_2_O_2_CO_3_ facilitated by the composite structure significantly improved the electrical conductivity and optimized the intermediate adsorption behavior, resulting in enhanced activity, selectivity, and durability for eCO_2_RR toward formate production. Subsequent in situ Raman tests and postcatalysis characterizations revealed that the reduction reconstruction of Bi-based materials during the eCO_2_RR process can be effectively resisted through the regulation of an alkaline local environment, which maintains the β-Bi_2_O_3_/Bi_2_O_2_CO_3_ composite structure.

Figure 1a shows a facile two-step method for synthesizing BO/BOC. First, a Bi-based precursor (see Figures S1–S3 for its structural characterization) was synthesized through a solvothermal reaction at 140 °C for 24 h using Bi(NO_3_)_3_·5H_2_O as the Bi source. This precursor was further calcinated in air at 240 °C, during which the decomposition of the organic and nitrate components took place to form Bi_2_O_2_CO_3_ (Figure S4), and subsequent continuous calcination finally led to a β-Bi_2_O_3_/Bi_2_O_2_CO_3_ composite with nanoflower morphology. The scanning electron microscopy (SEM) image illustrates the nanosheet-assembled nanoflower structure with a diameter of ∼200 nm for BO/BOC (Figure 1b). The transmission electron microscopy (TEM) image reveals ultrathin thickness and ∼50 nm diameter of the nanosheets (Figure 1c). The high-resolution TEM (HRTEM) images of BO/BOC (Figure 1d and Figure S5a) display regions with distinct lattice orientations, clearly suggesting the composite structure. Closer observation (Figure 1e and Figure S5b) finds lattice fringes of 0.295 nm in the purplish-red region, corresponding to the (013) facet of Bi_2_O_2_CO_3_,^42−44^ and 0.386 nm in the orange region, relating to the (110) facet of β-Bi_2_O_3_.^45,46^ TEM images from another region also show the composite structure of BO/BOC (Figures S6 and S7). Geometric phase analysis (GPA) was applied to analyze lattice strain in the sample,^47^ showing clear tensile strain at the top and compressive strain at the bottom (Figure 1f). Additionally, fast Fourier transform (FFT) analysis revealed two sets of distinct facets of Bi_2_O_2_CO_3_ and β-Bi_2_O_3_ in the sample (Figure 1g,h). The high-angle annular dark field-scanning TEM (HAADF-STEM) image and corresponding X-ray energy dispersive spectrometry (EDS) mappings of BO/BOC highlight the homogeneous dispersion of Bi, O, and C elements along the nanosheets (Figure 1i–l). These results clearly demonstrate the dual-phase composite and the successful integration of β-Bi_2_O_3_ and Bi_2_O_2_CO_3_.

**Figure 1:**
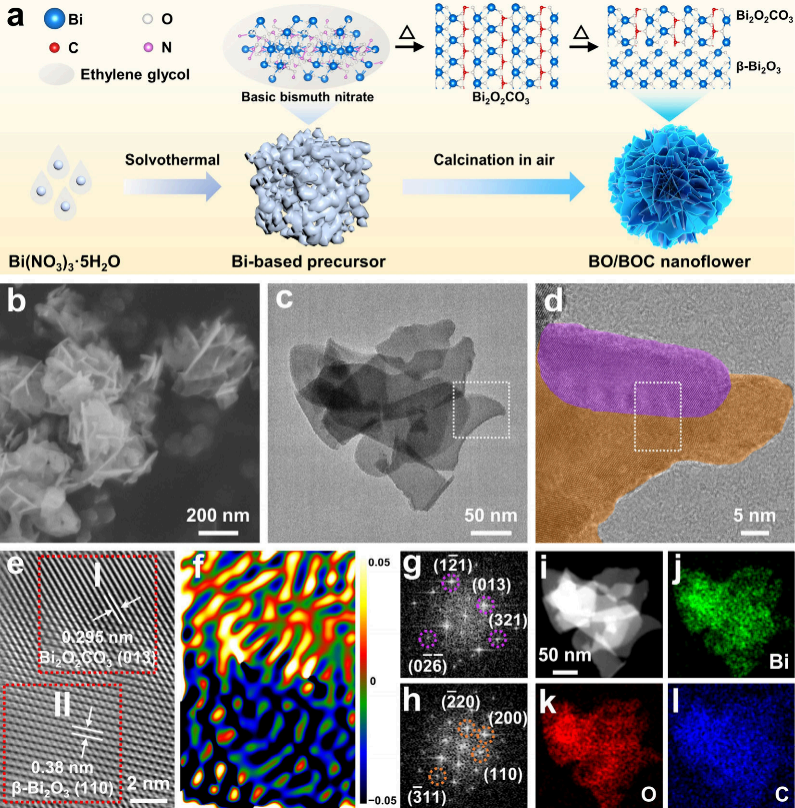
(a) Schematic illustration of the synthesis of BO/BOC. (b) SEM image and (c, d) TEM images with (c) low and (d) high magnifications of BO/BOC. The purplish-red and orange regions in panel d represent Bi_2_O_2_CO_3_ and β-Bi_2_O_3_, respectively. (e) Inverse Fourier transform result and (f) corresponding *E*_*xy*_ GPA of selected region in panel d. (g, h) FFT results of (g) region I and (h) region II in panel e. The purplish-red and orange circles represent the crystal facets of Bi_2_O_2_CO_3_ and β-Bi_2_O_3_, respectively. (i) HAADF-STEM image of BO/BOC and (j–l) corresponding EDS elemental mapping results of (j) Bi, (k) O, and (l) C.

To understand the formation mechanism of the β-Bi_2_O_3_/Bi_2_O_2_CO_3_ sample, a series of control experiments were conducted. First, the exploration of the effect of calcination temperature (Figures S8–S10) indicate that the calcination temperature of 240 °C is critical to obtain β-Bi_2_O_3_/Bi_2_O_2_CO_3_ with a nanosheet-assembled nanoflower morphology. Then, subsequent exploration of calcination time revealed unchanged morphology of BO/BOC (Figure S11) while the β-Bi_2_O_3_ content increased with prolonged calcination time (Figure S12). By refinement of X-ray diffraction (XRD) data (Figure S13), the mass ratios of β-Bi_2_O_3_/Bi_2_O_2_CO_3_ in BO/BOC calcinated at 240 °C for 3, 5, and 7 h were determined to be 27:73, 49:51, and 55:49, respectively, indicating that the ratio can be easily controlled by adjusting the calcination time.

For a comprehensive analysis of the structural characteristics of BO/BOC, β-Bi_2_O_3_ nanosheets (BO NS) and BOC nanosheets (BOC NS) (see Figures S14–S16 for their structural characterization) were synthesized for comparison. Figure S17 shows the distinct Raman peaks of BO/BOC compared to BO NS and BOC NS, illustrating the formation of a new β-Bi_2_O_3_/Bi_2_O_2_CO_3_ composite rather than a simple mixture of these two components. The Bi 4f X-ray photoelectron spectroscopy (XPS) signal in Figure S18a shows a 0.8 eV positive shift in the Bi^3+^ 4f_7/2_ binding energy for BO/BOC compared to BO NS and a 0.2 eV negative shift relative to BOC NS, indicating electron transfer from β-Bi_2_O_3_ to Bi_2_O_2_CO_3_ in BO/BOC. Additionally, BO/BOC exhibits 0.6 eV positive shifts for both Bi–O and CO_3_^2–^ species in O 1s spectra (Figure S18b) and a 0.2 eV negative shift for the C–O=C species in the C 1s spectra (Figure S18c) compared to BOC NS, further supporting this electron transfer. The ultraviolet photoelectron spectroscopy (UPS) spectra (Figure S19a) showed that the work function (Φ) values of BO/BOC, BO NS, and BOC NS are 4.99, 4.73, and 5.24 eV, respectively (Figure S19b). A lower Φ value suggests a smaller energy barrier for electrons escape.^48,49^ Thus, the combined XPS and UPS results enhance the understanding of directional electron transfer from β-Bi_2_O_3_ to Bi_2_O_2_CO_3_ in BO/BOC (Figure S20). Additionally, Kelvin probe force microscopy (KPFM) was employed to detect the surface potential distribution of materials,^50^ which exhibits a characteristic edge-to-center gradient (Figure S21), clearly demonstrating the presence of a unidirectional built-in electric field from edge to center for BO/BOC.^51^ Such difference in surface potential could be ascribed to the different work functions of the respective parts.^52^ The unique electronic structure and directional electron transfer effect could be due to the optimized the band gaps of both β-Bi_2_O_3_ to Bi_2_O_2_CO_3_ when the composite is formed, which highly promotes the electron migration rates and could benefit the electrochemical performance.

The eCO_2_RR performance of BO/BOC with different β-Bi_2_O_3_/Bi_2_O_2_CO_3_ mass ratios was first measured and compared. The results show that BO/BOC with a mass ratio of ∼1:1 (BO/BOC-5h) exhibits a higher current density (Figure S22a), higher faradaic efficiency for formate (FE_formate_, Figure S22b), and higher partial current density of formate (*J*_formate_) (Figure S22c) than BO/BOC-3h and BO/BOC-7h. These findings underscore that the optimal β-Bi_2_O_3_/Bi_2_O_2_CO_3_ mass ratio significantly influences the eCO_2_RR performance of BO/BOC, as evidenced by its lower *R*_ct_ value (Figure S22d and Table S1) and higher electrical double-layer capacitance (*C*_dl_) (Figure S23) indicating a larger electrochemically active surface area for BO/BOC.^53^

Then, the eCO_2_RR performance of BO/BOC was compared to those of BO NS, BOC NS, and a mechanical mixture of BO NS and BOC NS (mixed BO/BOC NS). Linear sweep voltammetry (LSV) curves reveal that BO/BOC achieves a significantly higher current density than mixed BO/BOC NS, BO NS, and BOC NS (Figure 2a). The high-performance liquid chromatography (HPLC) and gas chromatography (GC) results in Figures S24–S26 indicate that HCOOH, CO, and H_2_ are the main products of the catalysts. The FEs of the products for BO/BOC, mixed BO/BOC NS, BO NS, and BOC NS are presented in Figure 2b and Figure S27. BO/BOC exhibits the highest selectivity toward formate, achieving over 94% FE_formate_ across a wide potential window (from −0.7 to −1.1 V) and a maximum FE_formate_ of 96.6% at −1.0 V (Figure 2c), whereas mixed BO/BOC NS, BO NS and BOC NS have lower FE_formate_ under 65%. Notably, the FE_formate_ at −1.0 V of BO/BOC is 1.72-, 1.70- and 1.98-fold higher than that of BO/BOC NS (56.1%), BO NS (56.7%), and BOC NS (48.8%), respectively, indicating a significant enhancement in selectivity. Moreover, the *J*_formate_ for BO/BOC reaches 23.0 mA cm^–2^ at −1.0 V, which is 1.97-, 2.15-, and 2.47-fold higher than that of BO/BOC NS (11.7 mA cm^–2^), BO NS (10.7 mA cm^–2^), and BOC NS (9.33 mA cm^–2^) (Figure 2d). Cyclic voltammograms (CV) results (Figure S23b and Figure S28) show that BO/BOC exhibits a larger *C*_dl_ (3.95 mF cm^–2^) than mixed BO/BOC NS (3.26 mF cm^–2^), BO NS (2.42 mF cm^–2^), and BOC NS (3.04 mF cm^–2^) (Figure 2e), suggesting the highest number of active sites in BO/BOC to promote eCO_2_RR. Additionally, electrochemical impedance spectroscopy (EIS) results further revealed that the directional electron transfer in BO/BOC leads to improved overall electrical conductivity and reduced interfacial charge-transfer resistance (*R*_ct_, Figure 2f, Figure S29 and Table S1).

**Figure 2:**
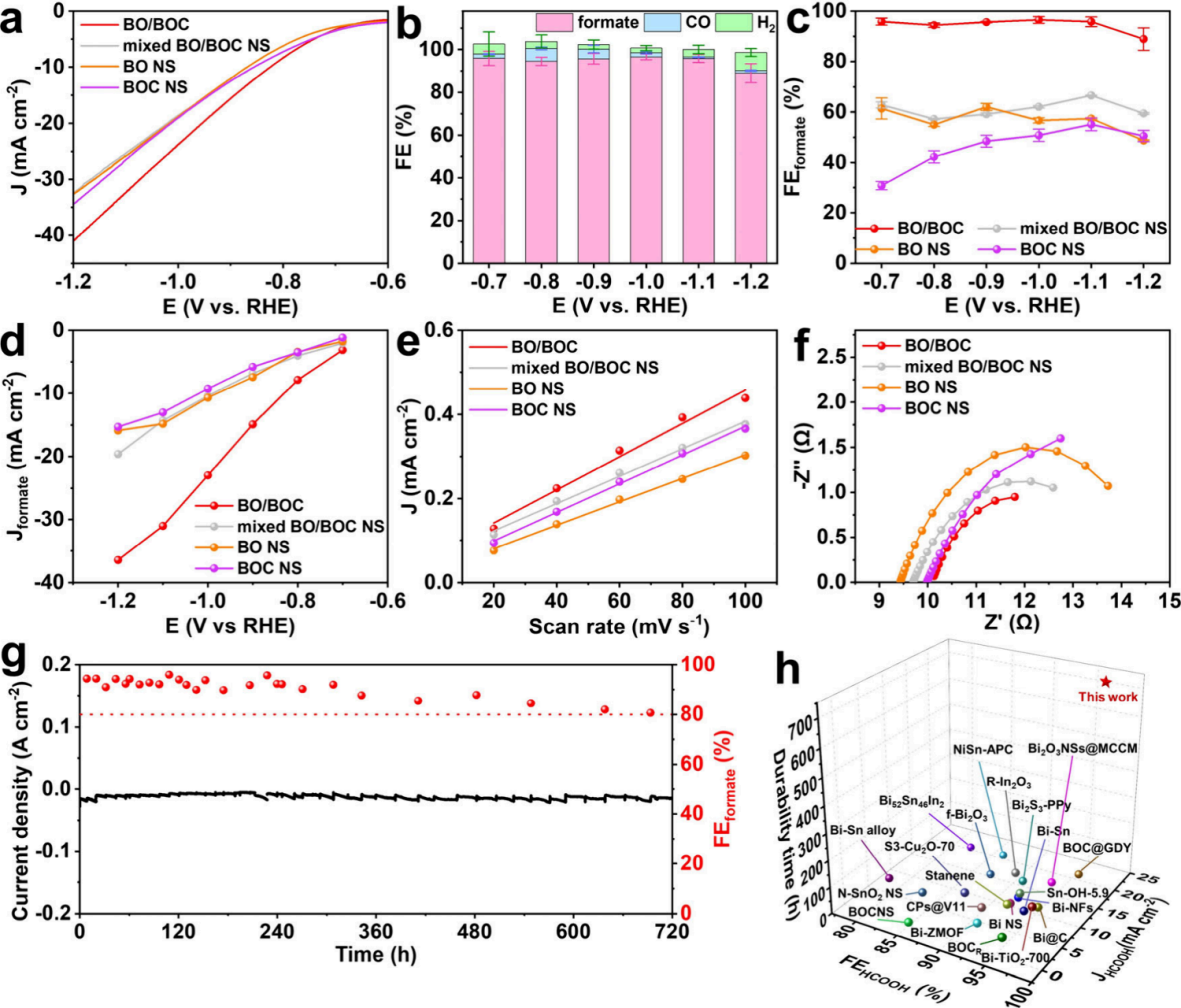
(a) LSV curves of BO/BOC, mixed BO/BOC NS, BO NS, and BOC NS in CO_2_-saturated 0.5 M KHCO_3_. (b) FE for the eCO_2_RR products of BO/BOC. The data were averaged over three repeated measurements with the standard deviations marked by pink error bars for formate, light blue error bars for CO, and light green error bars for H_2_. (c) FE for formate, (d) *J*_formate_, (e) capacitive current density versus scan rate, and (f) fitted EIS results of BO/BOC, mixed BO/BOC NS, BO NS, and BOC NS. The data in panel c were averaged over three repeated measurements with the standard deviations. (g) Stability test of BO/BOC at −1.0 V in CO_2_-saturated 0.5 M KHCO_3_. (h) Comparison of electrochemical CO_2_RR performance of BO/BOC for formate production to that of recently reported novel catalysts.

The durability of BO/BOC was evaluated through long-term eCO_2_RR at −1.0 V (Figure 2g). After 100 h of electrocatalysis, BO/BOC maintained a higher FE_formate_ (>92%) compared to BO/BOC-3h (90.2%) and BO/BOC-7h (88.7%) (Figure S30). Remarkably, it sustained a stable current density and FE_formate_ above 80% for 720 h during continuous electrolysis, demonstrating the exceptional durability of BO/BOC for eCO_2_RR. Notably, compared with recently reported Bi-based and other metal-based catalysts, BO/BOC shows improvements in FE_formate_, *J*_formate_, and especially durability (Figure 2h and Table S2).

In situ Fourier transform infrared spectroscopy (FTIR) spectroscopy was conducted to underscore the eCO_2_RR mechanism of the catalysts. As the working potential increased, all samples displayed two apparent peaks at ∼1280 and ∼1400 cm^–1^ (Figure 3a–d), corresponding to the CO_2_^•–^ radical and *OCHO intermediate, respectively.^54^ The presence of these two species during the eCO_2_RR process indicates that CO_2_ molecules adsorbed on the electrode surface are first activated to CO_2_^•–^ radicals and then converted to *OCHO.^55^ Interestingly, the intensities of these two species continuously increased for BO/BOC with increased potential, while mixed BO/BOC NS, BO NS, and BOC NS show trends of initial increase followed by a decrease (Figure 3e). This phenomenon accounts for the high FE_formate_ of BO/BOC across a wide potential range.

**Figure 3:**
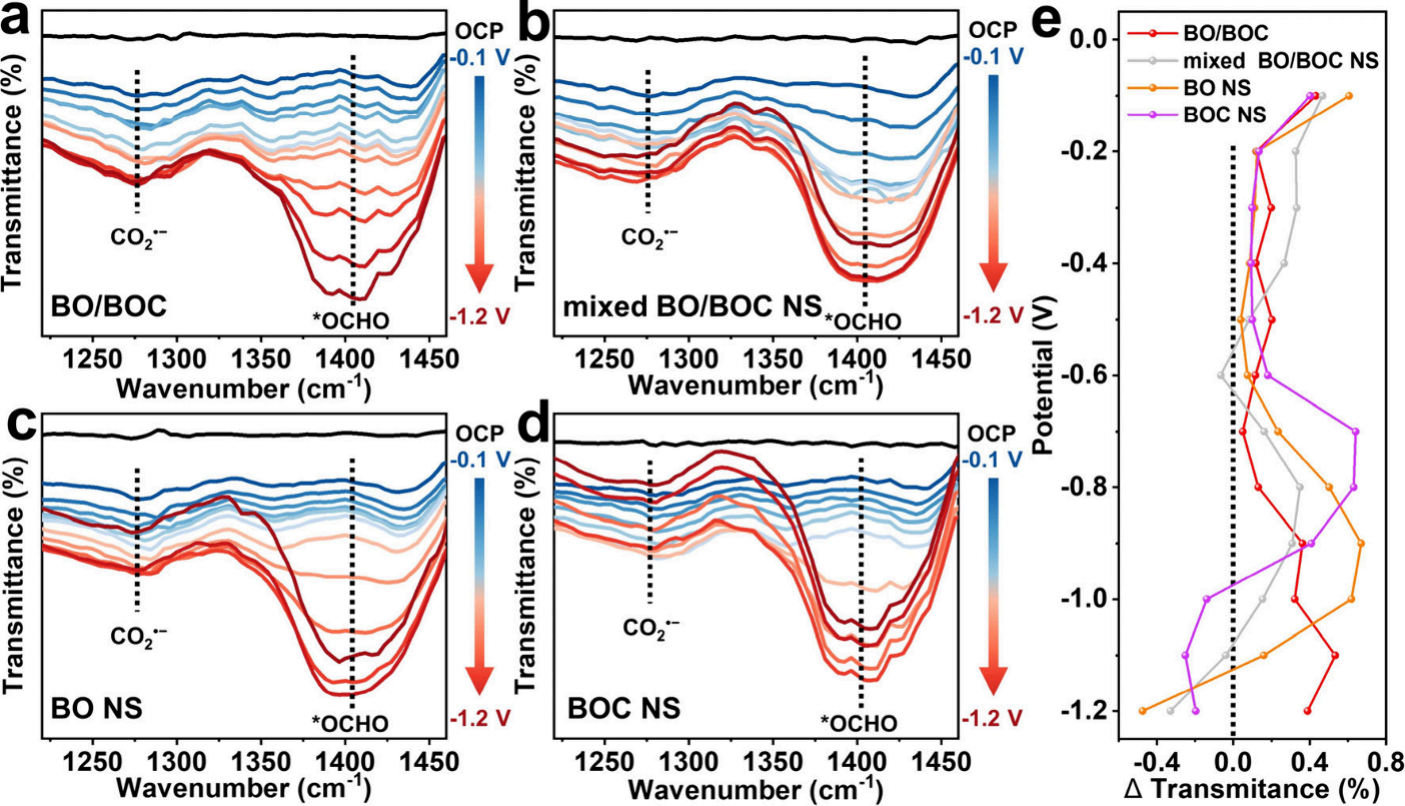
In situ FTIR spectra of (a) BO/BOC, (b) mixed BO/BOC NS, (c) BO NS, and (d) BOC NS. (e) Difference in FTIR intensity for *OCHO of each applied potential compared to the last measured potential for BO/BOC, mixed BO/BOC NS, BO NS, and BOC NS.

In situ Raman spectroscopy was performed to investigate the structural evolution of catalysts during the eCO_2_RR process. For BO/BOC, the increase in the working voltage leads to the gradual disappearance of the Bi–O peaks around 310 and 460 cm^–1^, while those in the 80–210 cm^–1^ range merge into a broad Bi–O peak around 120 cm^–1^ (Figure 4a). This may result from the lattice fusion of β-Bi_2_O_3_ and Bi_2_O_2_CO_3_, leading to the embedding of originally characteristic Bi–O peaks into a new Bi–O bond. For mixed BO/BOC NS, BO NS, and BOC NS, the typical Bi–O peaks at 118, 305, and 462 cm^–1^ for β-Bi_2_O_3_ and the characteristic Bi–O peak at 154 cm^–1^ for Bi_2_O_2_CO_3_ gradually vanish, while a new peak at 97 cm^–1^ corresponding to the Bi–Bi bond in metallic Bi^56^ emerges as the working voltage increases (Figure 4b–d). Upon returning to the open circuit potential (OCP), the Raman spectra of the catalysts remain unchanged from those at −1.2 V, indicating irreversible structural change and the accomplishment of the reconstruction process. After in situ Raman testing, BO/BOC retains its original color, while the originally white or yellow samples of mixed BO/BOC NS, BO NS, and BOC NS turn black (Figure S31), suggesting reduction to nanosized metallic Bi. These findings unequivocally demonstrate the propensity of β-Bi_2_O_3_, Bi_2_O_2_CO_3_, or a simple mixture of the two to undergo reduction to metallic Bi, contrasting with the resistance of BO/BOC to such reduction processes. CV tests provide more insights for the resistance to the reduction reconstruction of BO/BOC. The CV curves reveal distinct redox peaks for all catalysts (Figure S32), consistent with prior reports.^57^ Notably, BO/BOC exhibits a significantly weaker and more negative reduction peak compared to mixed BO/BOC, BO NS, and BOC NS, indicating a higher reduction activation energy that leads to more difficult reduction during the eCO_2_RR process.^58,59^

**Figure 4:**
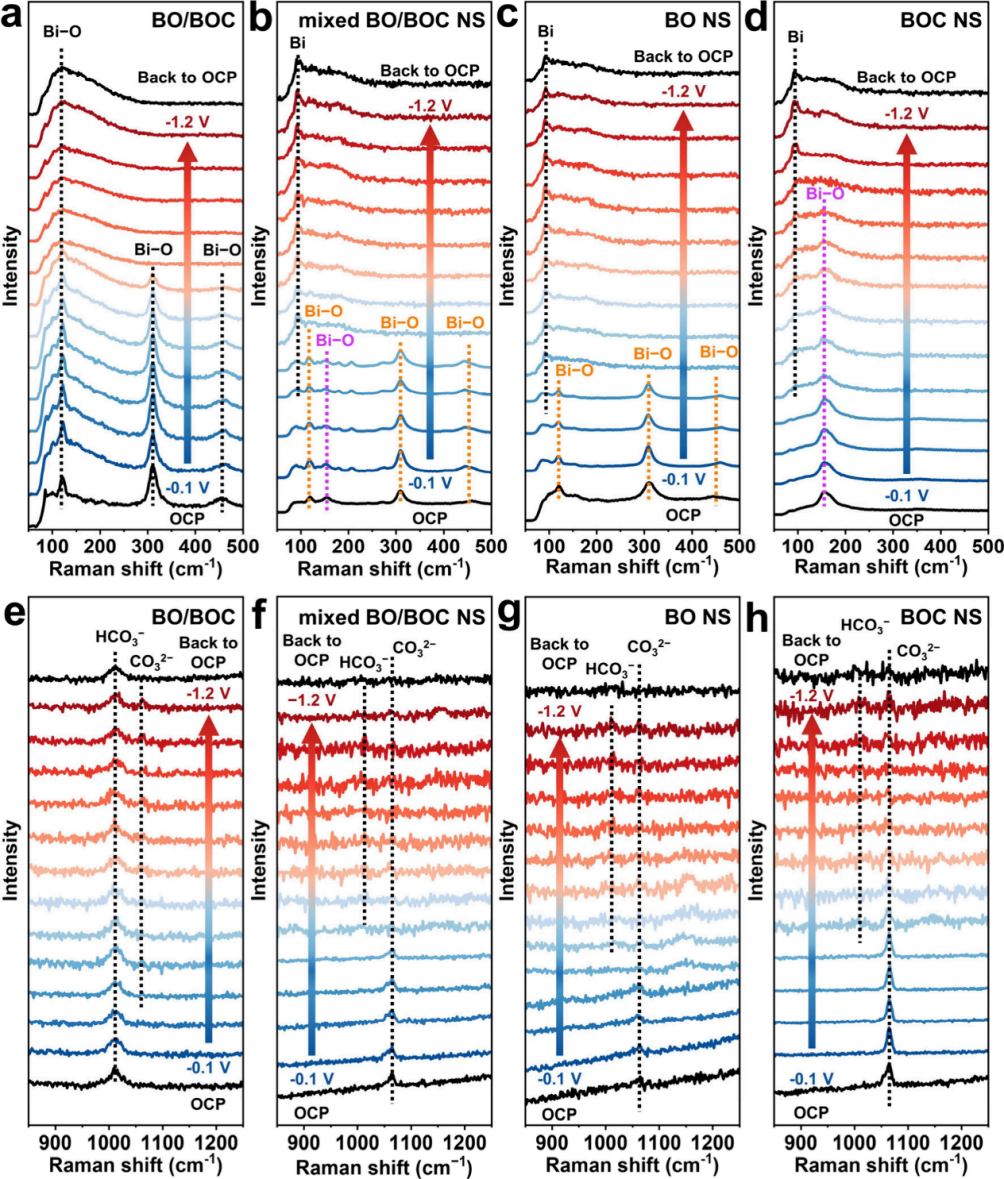
In situ Raman spectra of (a) BO/BOC, (b) mixed BO/BOC NS, (c) BO NS, and (d) BOC NS at the range of 50–500 cm^–1^. In situ Raman spectra of (e) BO/BOC, (f) mixed BO/BOC NS, (g) BO NS, and (h) BOC NS at the range of 850–1250 cm^–1^.

The Raman spectra at high wave numbers provide deeper insight into the reconstruction. At OCP, a peak at 1012 cm^–1^ attributed to the HCO_3_^–^ species in the electrolyte^60^ can be initially identified for BO/BOC (Figure 4e). As the working voltage increases, a peak at 1069 cm^–1^ assigned to CO_3_^2–^ species^61^ appears and intensifies. The decreased HCO_3_^–^/CO_3_^2–^ ratio suggests a more alkaline microenvironment, which can facilitate the regeneration of the Bi–O structure and thus resist reduction during eCO_2_RR.^62^ Conversely, increased HCO_3_^–^/CO_3_^2–^ ratios are noted for mixed BO/BOC NS, BO NS, and BOC NS (Figure 4f–h), indicating a more acidic environment. Hence, the ability to regulate the local environment through constructing the β-Bi_2_O_3_/Bi_2_O_2_CO_3_ composite plays a pivotal role in effectively resisting the reduction reconstruction of Bi-based materials.

Postcatalysis characterizations were conducted to examine the structures of BO/BOC after durability tests (denoted as BO/BOC-48h and BO/BOC-720h for the sample after 48 and 720 h of durability test). TEM images show unchanged nanosheet morphologies for both BO/BOC-48h BO/BOC-720h, although numerous pores several nanometers in size appear on the nanosheet (Figure S33a,c). Time-dependent inductively coupled plasma atomic emission spectroscopy (ICP-AES) analysis showed that the Bi element content in the electrolyte gradually increased over the first 18 h, followed by a sudden decrease at the 24 h (Figure S34), indicating that Bi leaching is almost accomplished within 24 h. The pH after 24 h of eCO_2_RR was slightly reduced to 6.80, indicating that the leaching of Bi was driven by the structural evolution by eCO_2_RR rather than the pH. HRTEM observations illustrate the maintenance of the β-Bi_2_O_3_/Bi_2_O_2_CO_3_ composite after durability tests, despite a decrease in the particle size (Figure S33b,d). XRD patterns for BO/BOC samples after durability tests also displayed obvious characteristic peaks corresponding to β-Bi_2_O_3_ and Bi_2_O_2_CO_3_ (Figure S35), confirming the preservation of the composite structure. Notably, the XRD peak signal of Bi_2_O_2_CO_3_ shows an obvious decrease, demonstrating the decomposition of the Bi_2_O_2_CO_3_ component during the eCO_2_RR process. Subsequent XPS analysis indicated gradual shifts of Bi^3+^ 4f_7/2_, Bi–O, and CO_3_^2–^ species to higher binding energy in the Bi and O XPS signals (Figure S36a,b), as well as the gradual decrease in the areas of CO_3_^2–^ and C–O=C species in O and C XPS signals (Figure S36b,c). These results further support the gradual decomposition of the Bi_2_O_2_CO_3_ component in BO/BOC, which leads to a transformation from a more BOC-like structure to a more Bi_2_O_3_-like structure for BO/BOC and therefore a decrease in the activity for eCO_2_RR. After the durability tests, BO/BOC-48h (3.49 eV) and BO/BOC-720h (3.40 eV) displayed significantly lower Φ values compared to that of pristine BO/BOC (3.99 eV) (Figure S37), indicating a more efficient directional electron transfer in BO/BOC after durability tests. Combining the in situ Raman results and the postcatalysis characterizations, the controllable reconstruction mechanism can be clearly understood, as illustrated in Figure S38. As the eCO_2_RR process progresses, the components in the original β-Bi_2_O_3_/Bi_2_O_2_CO_3_ composite leach, creating abundant pores on the BO/BOC nanosheets as the TEM results in Figure S33a show. Subsequently, the nanosheets undergo a reconstruction and lattice fusion process, leading to the disappearance of initial characteristic Raman peaks and formation of new Bi–O bonds, as evidenced by in situ Raman results in Figure 4. With the continuous structural evolution proven by XRD and XPS spectra in Figures S35 and S36, a decrease of Bi_2_O_2_CO_3_ content takes place, but the β-Bi_2_O_3_/Bi_2_O_2_CO_3_ composite structure is maintained even after 720 h of durability testing.

In summary, a novel β-Bi_2_O_3_/Bi_2_O_2_CO_3_ composite was designed with high resistance to reduction reconstruction due to its ability to regulate a more alkaline microenvironment that facilitates the formation of new Bi–O bonds during the eCO_2_RR process. Such a controllable reconstruction for BO/BOC allows for the preservation of the β-Bi_2_O_3_/Bi_2_O_2_CO_3_ composite and its directional electron transfer after eCO_2_RR. By integration of the benefits of both β-Bi_2_O_3_ and Bi_2_O_2_CO_3_ components, the resulting material exhibited enhanced activity and selectivity and remarkably durability in eCO_2_RR. This work sheds new light on designing novel high-performance nanomaterials and unraveling the reconstruction mechanism toward eCO_2_RR and other practical applications.

## Supporting Information Available

Experimental details, SEM, XRD, FTIR, TGA-DTG, TEM, EDS, Raman, XPS, KPFM, HPLC, GC, and electrochemical results (PDF)
