# Supplementary material for: Controllable Reconstruction of β-Bi2O3/Bi2O2CO3 Composite for Highly Efficient and Durable Electrochemical CO2 Conversion
Source: Nano Lett. 2025 Apr 9;25(16):6548–55. doi: 10.1021/acs.nanolett.5c00417 (PMC12023034; doi:10.1021/acs.nanolett.5c00417)
Supplement: Supplementary file 1 — nl5c00417_si_001.pdf [file nl5c00417_si_001.pdf]

Controllable Reconstruction of β-Bi_2O_3/$Bi_2O_2CO_3$ Composite for Highly Efficient and Durable Electrochemical CO_2 Conversion

Yuxuan Xiao,[1] Di Liu,[1] Jiao Yang,[1] Jinxian Feng,[1] Wenhao Gu,[1] Lulu Qiao,[1] Weng Fai IP,[2] and Hui Pan*[12]

[1]Institute of Applied Physics and Materials Engineering, University of Macau, Macao SAR, China.

[2]Department of Physics and Chemistry, Faculty of Science and Technology, University of Macau, Macao SAR, China.

* To whom correspondence should be addressed.

Email: huipan@um.edu.mo (H. P.)

Experimental details

***Synthesis of Bi-based precursor*:** 0.1 g of $Bi(NO_3)_3·5H_2O$ and 1 mL of ammonia water were dissolved in 40 mL of ethylene glycol and 40 mL of benzyl alcohol with vigorous stirring for 30 min. Then, the resulting solution was transferred to a Teflon-lined stainless steel autoclave and maintained at 140 °C for 24 h. The Bi-based precursor was obtained after centrifugation at 5,000 rpm for 5 min, washing with methanol for 3 times, and drying at 70 °C overnight.

***Synthesis of BO/BOC*:** BO/BOC was obtained by calcination of Bi-based precursor in a tubular furnace at 240 °C for 5 h with a heating rate of 4 °C min^{-1} in air. Similar procedures were followed for the synthesis of BO/BOC-3 h and BO/BOC-7 h, with the only variation being the adjustment of the calcination time to 3 h or 7 h.

***Synthesis of BO NS*:** In a typical synthesis, 0.29 g of $Bi(NO_3)_3·5H_2O$ was first dissolved in a 40 mL of ethylene glycol with vigorous stirring for 30 min. Then, 8 mL of 2.25 mg mL^{-1} $NaBH_4$ solution was added dropwise within 15 min. After centrifugation at 12,000 rpm for 3 min and washing with methanol for 3 times, Bi nanosheets were obtained by drying at 70 °C overnight. Finally, the BO NS were obtained by calcination of Bi nanosheets in a tubular furnace at 150 °C for 5 h with a heating rate of 4 °C min^{-1} in air.

***Synthesis of BOC NS*:** 0.5 g of $Bi(NO_3)_3·5H_2O$ (dissolved in 20 mL of deionized water) and 0.3 g of urea (dissolved in 20 mL of alcohol) were mixed together by magnetic stirring. After stirring for 30 min, the above mixture was transferred to a Teflon-lined stainless steel autoclave and maintained at 90 °C for 4 h. The resultant BOC NS was obtained after centrifugation at 5,000 rpm for 5 min, washing with methanol for 3 times, and drying at 70 °C overnight.

***Synthesis of mixed BO/BOC*:** The mixed BO/BOC was obtained by combining 0.1 g of BO NS and 0.1 g of BOC NS in 50 mL of de-ionized water with vigorous stirring for 2 h, followed by centrifugation at 8,000 rpm for 10 min and drying at 70 °C overnight.

Preparation of Working Electrodes: 10 mg of catalyst powder was dispersed in a solution containing 880 μL of isopropanol, 100 μL of de-ionized water and 20 μL of Nafion resin solution with the help of ultrasonication to form a catalyst ink. Then, 100 μL of the resulting catalyst ink was dropped onto the carbon paper (1 × 1 cm), dried under an infrared lamp for 30 min to totally evaporate the solvent, and used as the working electrode.

***Physical characterization*:** TEM images, HAADF-STEM images, and EDS analyses were collected using a FEI Talos microscope. SEM micrographs were taken using a field-emission scanning electron microscope (Carl Zeiss). XRD patterns were collected using an X-ray diffractometer (Rigaku) equipped with Cu Kα radiation. The XRD refinement was conducted

by the MDI-Jade software. By fitting the XRD pattern, each phase can be separated according to the standard PDF cards, and the mass ratio of each phase can be calculated accordingly by the MDI-Jade software. TGA-DTG curves were recorded on a Shimadzu TGA-51 thermogravimetric analyzer. XPS and UPS measurements were performed on an ESCALAB 250Xi (Thermo Fisher, USA). The Φ value was calculated as the difference between the intercept of the dashed line with the horizontal coordinate of the UPS spectrum and the photonic energy of He I (21.22 eV). KPFM measurement was conducted on a Bruker FastScanBio-Icon atomic force microscope system. In-situ Raman spectra were obtained by a micro Raman system (Horiba LabRAM HR Evolution) under ambient conditions at an excitation line of 532 nm, with 50% power ratio and the acquisition time of 5 s. In-situ FTIR spectra were obtained by a Shimadzu IRXross FTIR Spectrophotometer through reflection method. CO_2-saturated 0.5 M $KHCO_3$ was used as the electrolyte for the in-situ characterizations. The electrolyte was kept flowing by a peristaltic pump for in-situ Raman test, and the CO_2 flow rate was 20 sccm. The Bi element content in electrolyte was detected via ICP-AES (Leeman Laboratories Inc.).

***Electrochemical measurements*:** The electrochemical measurements were carried out at room temperature in a Nafion 117 membrane separated H-type cell with 0.5 M $KHCO_3$ solution as electrolyte. The as-prepared carbon paper was used as working electrode, a commercial ruthenium-iridium titanium plate as the counter electrode, and a saturated calomel electrode (SCE) as a reference electrode. Prior to the electrochemical measurements, CO_2 was bubbled into the electrolyte for at least 30 min. The pH of the CO_2-saturated 0.5 M $KHCO_3$ electrolyte was measured to be 7.26. The cyclic voltammetry (CV) measurements were applied at a sweep rate of 50 mV s^{-1} for 50 cycles to obtained a steady curve. Linear sweep voltammetry (LSV) tests were performed with a scan rate of 20 mV s^{-1} was repeated seven times, and the last one was plotted. During the electrolysis, the cathode electrolyte was stirred at a rate of 500 rpm with a magnetic stirrer. The electrochemical impedance spectroscopy (EIS) tests were conducted from 100 kHz to 0.1 Hz at −1.0 V. Double-layer capacitance (C_{dl}) was measured by conducting CV between 0.23 and 0.33 V to evaluate the ECSA of catalysts. The double charge current density was plotted against the scan rates, and the linear fitting gave the Cdl value. All the electrochemical data were obtained without iR-compensation. All the potentials are presented with reference to the reversible hydrogen electrode (RHE) according to E (V vs RHE) = E (V vs SCE) + 0.242 + 0.0592 × pH.

***Product calculation*:** The gas products were quantified by a gas chromatography (Agilent 7890B). The FE of the gas products was calculated using the following equation:

$$FE_g(\%)=\frac{Q_g}{Q_{total}}\times100\%=\frac{N_g\times F\times v\times V_g/V_m}{I_{total}}\times100\%$$

where N_g and V_g represent the number of electrons transferred (N_g = 2) and the volume concentration of gas species generated, respectively. F is Faraday's constant (96485 C mol^{-1}), v is CO_2 flow rate (10 mL min^{-1}), V_m is the molar volume of gas (24 L mol^{-1}), and I_{total} is the steady-state current.

The liquid products were analyzed using a high-performance liquid chromatography (Agilent 1290 Infinity II) according to the calibration curve. The FE of formate was calculated as follows:

$$FE_{formate}(\%)=\frac{Q_{formate}}{Q_{total}}\times100\%=\frac{N_{formate}\times V_{formate}\times F}{I_{total}\times t}\times100\%$$

where $N_{formate}$ and $V_{formate}$ are the number of electrons transferred to produce formate ($N_{formate}$ = 2) and the amount of formate generated, respectively. t (s) is the electrolysis time.

Supporting Data

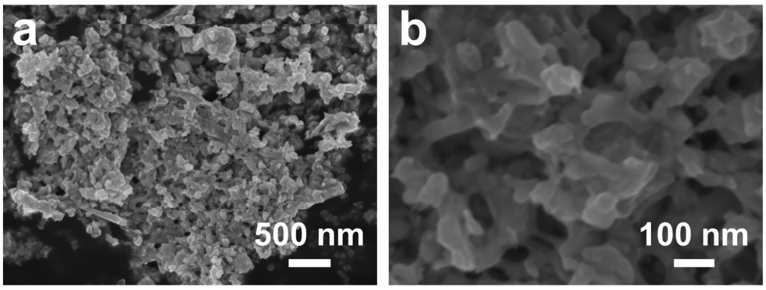

Figure S1. SEM images of the Bi-based precursor with (a) low and (b) high magnifications.

The obtained Bi-based precursor displays a three-dimensional structure with fused nanoparticles.

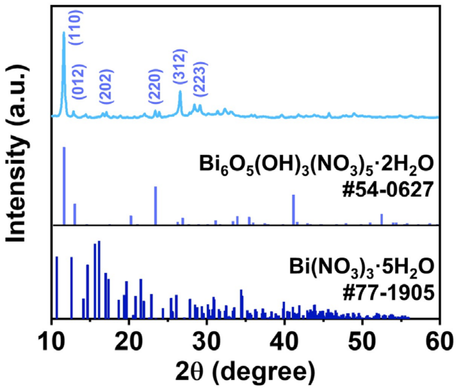

Figure S2. XRD pattern of the Bi-based precursor.

The crystal structure of the Bi-based precursor significantly differs from $Bi(NO_3)_3{\cdot}5H_2O$, but is close to basic bismuth nitrate ($Bi_6O_5(OH)_3(NO_3)_5{\cdot}2H_2O$), possibly due to the hydrolysis of $Bi(NO_3)_3{\cdot}5H_2O$ during the solvothermal process.[1]

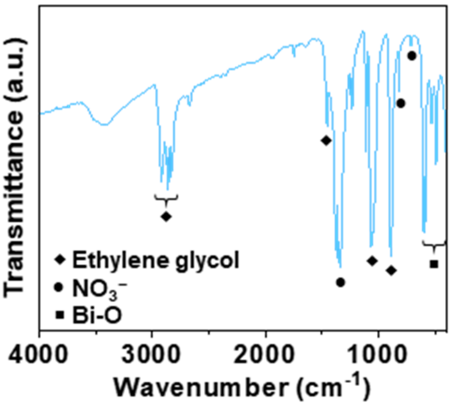

Figure S3. FTIR spectrum of the Bi-based precursor.

Fourier transform infrared spectroscopy (FTIR) result shows apparent signals of ethylene glycol groups (2920 cm^{-1} for C−H stretching, 1453 cm^{-1} for $-CH_2$ bending vibrations, and 891 and 1062 cm^{-1} for C−O stretching vibrations), NO_3^- groups (1339 cm^{-1} for the symmetric stretching, while 818 and 711 cm^{-1} for the asymmetric stretching modes of coordinated NO_3^- group) and

Bi−O groups (400 ~ 600 cm^{-1} for the stretching vibration of Bi−O bond) for the Bi-based precursor, indicating the coordination of BBN with ethylene glycol as reported previously.[2]

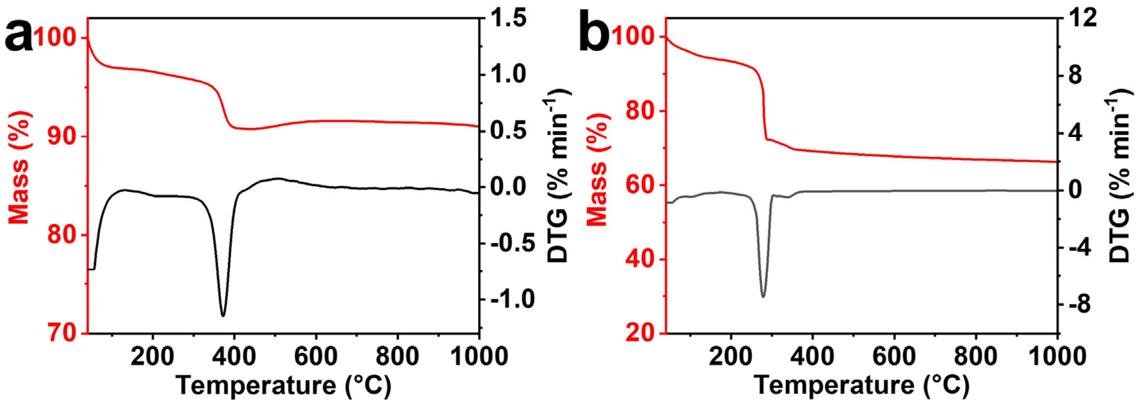

Figure S4. TGA-DTG curves of (a) BO/BOC and (b) Bi-based precursor.

The thermogravimetric analysis (TGA)-derivative thermogravimetry (DTG) curves of BO/BOC exhibit a significant mass loss between 210 and 420°C (Figure S4a), primarily due to the decomposition of $Bi_2O_2CO_3$ into Bi_2O_3, which is in accordance with prior study.[3] Specifically, the observed mass change (~4.47%) is less than the theoretical value for complete decomposition (8.63%), suggesting the presence of a $Bi_2O_2CO_3/Bi_2O_3$ composite structure within the BO/BOC matrix.

The TGA-DTG profiles of the bismuth-based precursor provide critical insights into the formation mechanism of BO/BOC (Figure S4b). Between 200 and 300°C, a massive mass loss of 22.0% is observed, which can be attributed to the pyrolysis of surface-adsorbed ethylene glycol (as evidenced by FTIR analysis in Figure S3) and the decomposition of the basic bismuth nitrate component in the precursor system.[4] Subsequently, from 300 to 400°C, a moderate mass decrease (2.75%) occurs, corresponding to the stepwise transformation of $Bi_2O_2CO_3$ into Bi_2O_3 as confirmed by the TGA-DTG data of BO/BOC (Figure S4a).

Based on the comprehensive TGA-DTG data, the formation process of BO/BOC can be elucidated as follows. At 240°C calcination, surface-bound organic species are thermally decomposed, while the bismuth nitrate precursor undergoes transformation into $Bi_2O_2CO_3$. Subsequent elevated temperature treatment induces further decomposition of $Bi_2O_2CO_3$, ultimately resulting in the formation of the dual-phase β-$Bi_2O_3/Bi_2O_2CO_3$ composite through a controlled thermal annealing process.

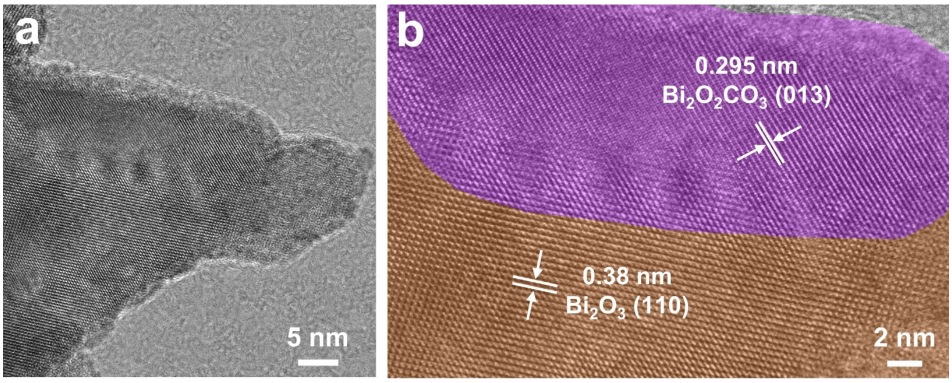

Figure S5. (a) Original TEM image in Fig. 1d. (b) Magnifying TEM image of (a).

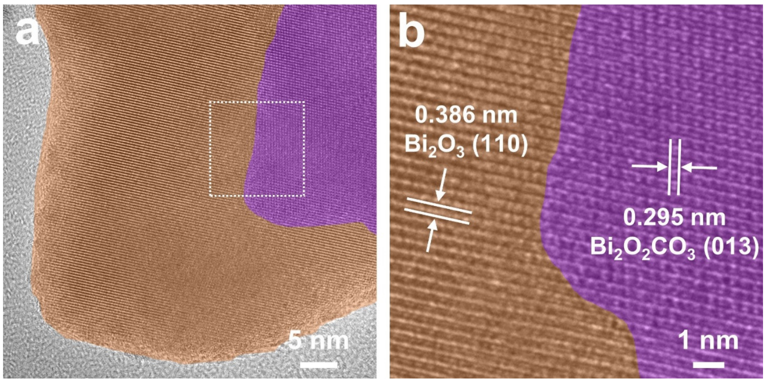

Figure S6. (a) TEM image and (b) HRTEM image of BO/BOC.

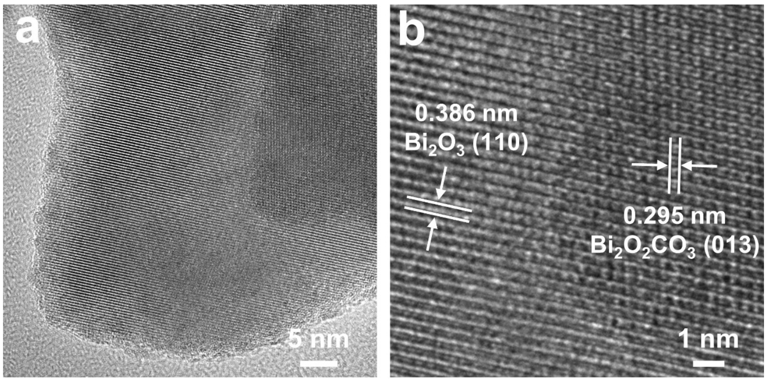

Figure S7. Original TEM images in Figure S6.

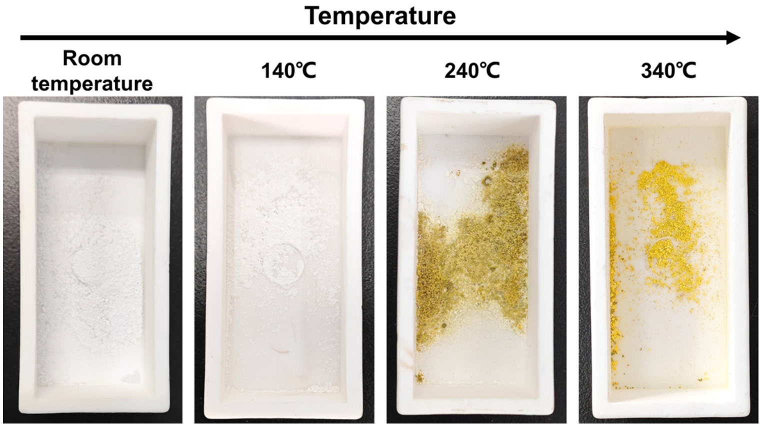

Figure S8. Optic photographs of the Bi-based precursors calcinated at different temperature.

The color of the sample changed from white to yellow as the temperature rises from 140°C to 340°C, signifying increased Bi_2O_3 content in the samples.

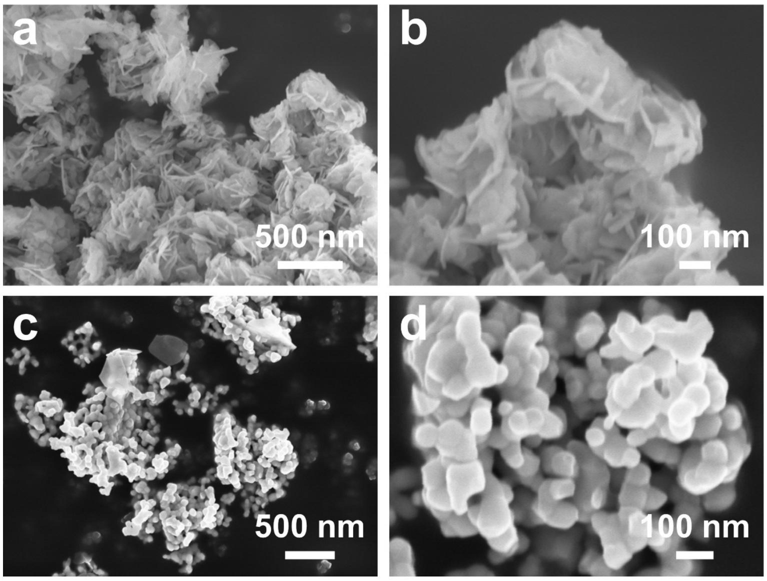

Figure S9. SEM images of the Bi-based precursors calcinated at (a,b) 140°C and (c,d) 340°C.

The sample calcinated at 140°C and 340°C displays the morphology of nanoflowers and nanoparticles, respectively.

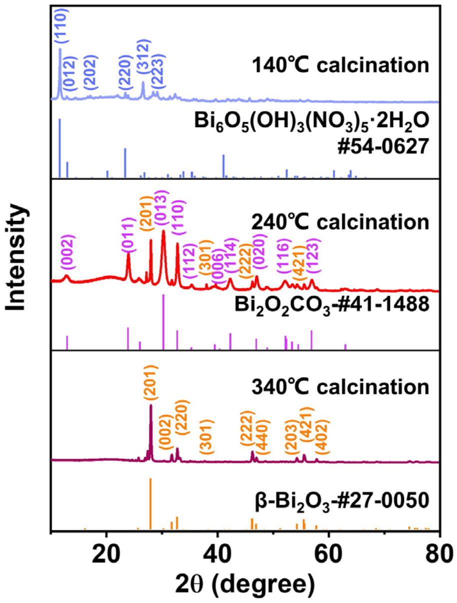

Figure S10. XRD patterns of the Bi-based precursors calcinated at 140°C, 240°C and 340°C.

With the increased calcination temperature, and the crystal structure of sample transfered from $Bi_6O_5(OH)_3(NO_3)_5{\cdot}2H_2O$ (at 140°C) to β-Bi_2O_3/$Bi_2O_2CO_3$ (at 240°C) and to pure β-Bi_2O_3 (at 340°C). Note that Bi_2O_3 in common has several phases, including a stable monoclinic α-phase (α-Bi_2O_3), metastable tetragonal β-phase (β-Bi_2O_3), a metastable body-centered cubic γ-phase (γ-Bi_2O_3) and a stable face-centered cubic δ-phase (δ-Bi_2O_3).[5] Among these, γ- and δ- are seldom reported for eCO_2RR. The β-phase has a higher CO_2 adsorption and activation energy than that of α-phase, which leads to facilitated intermediates transformation, and thus higher selectivity and activity for eCO_2RR.[6]

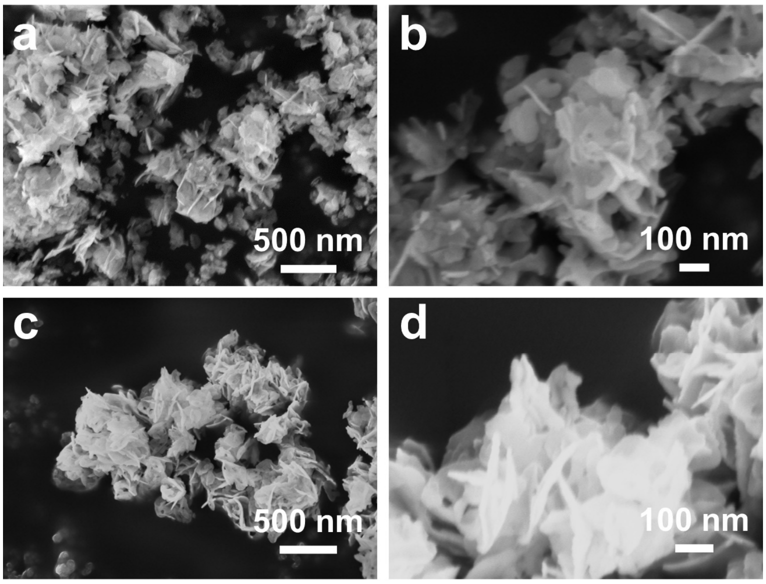

Figure S11. SEM images of BO/BOC calcinated at 240℃ for (a,b) 3 h and (c,d) 7 h.

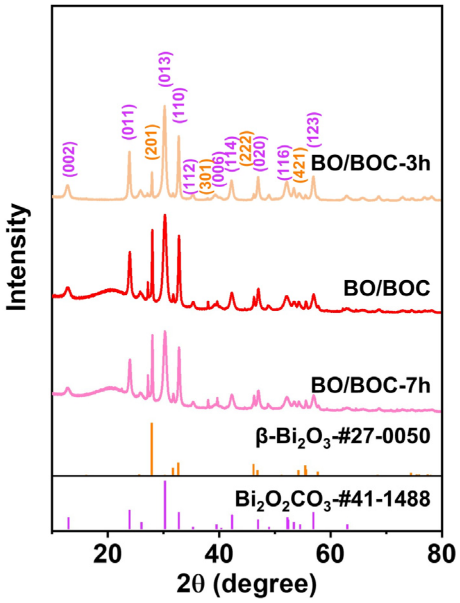

Figure S12. XRD patterns of BO/BOC calcinated at 240℃ for 3 h, 5 h and 7 h.

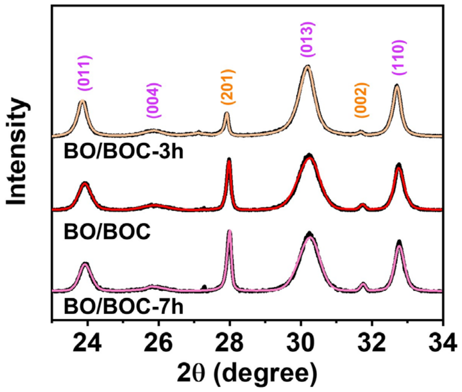

Figure S13. Refined XRD results of BO/BOC calcinated at 240℃ for 3 h, 5 h and 7 h. The black curves are the original data, while the colored curves are the refined data.

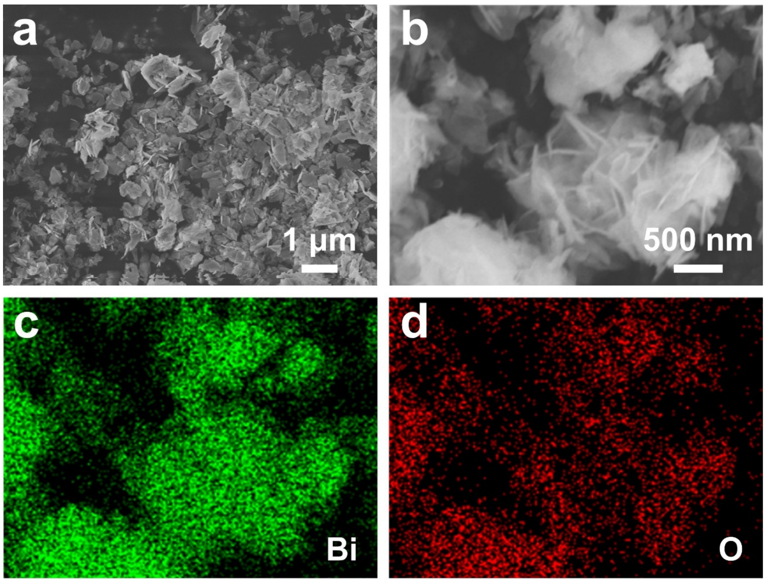

Figure S14. SEM images of BO NS with (a) low and (b) high magnifications. Corresponding EDS results showing signals of (c) Bi and (d) O.

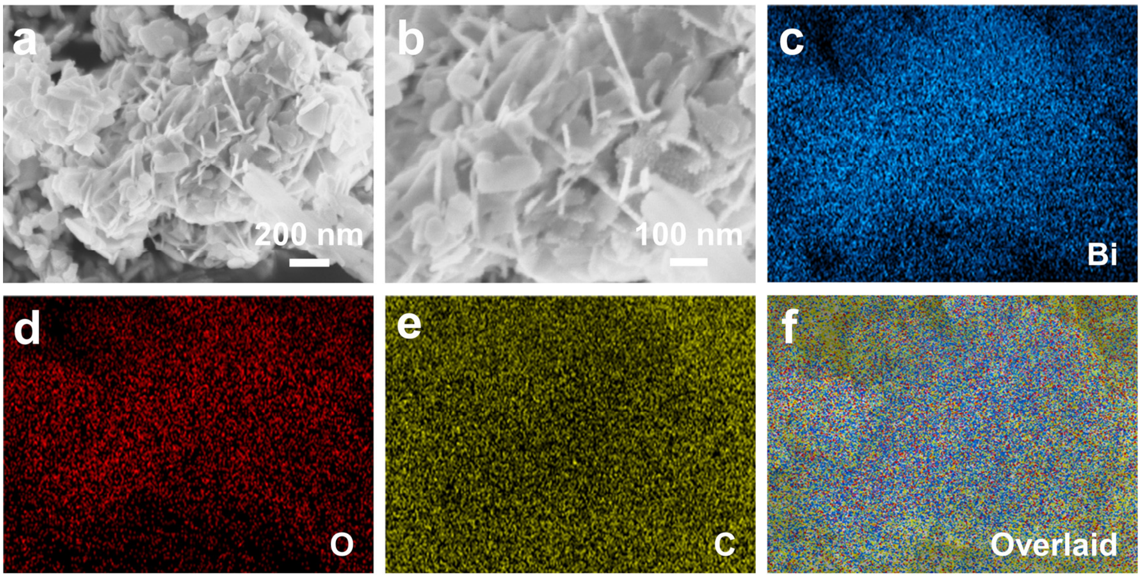

Figure S15. SEM images of BOC NS with (a) low and (b) high magnifications. EDS results showing signals of (c) Bi, (d) O, (e) C and (f) overlaid element mapping corresponding with (a).

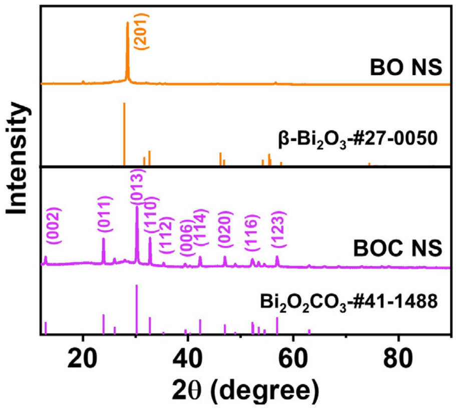

Figure S16. XRD patterns of BO/BOC, BO NS and BOC NS.

The XRD patterns of BO NS and BOC NS match well with the standard peaks of β-Bi_2O_3 and $Bi_2O_2CO_3$, indicating the pure phase of β-Bi_2O_3 and $Bi_2O_2CO_3$ in BO NS and BOC NS, respectively.

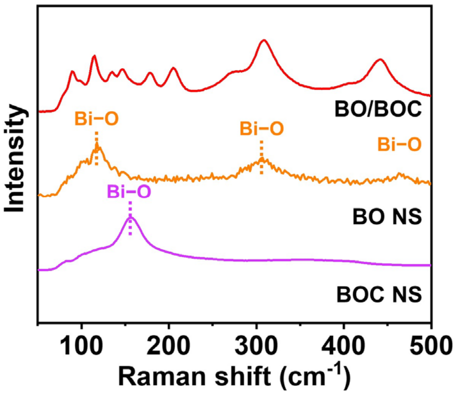

Figure S17. Raman spectra of BO/BOC, BO NS and BOC NS.

For bismuth oxide/subcarbonate-based materials, strong Raman bands appearing at wavenumbers <120 cm^{-1} can be assigned to the displacements of cationic $-Bi^{3+}-$ entities with regard to the surrounding oxide matrix, weaker bands appearing between 120 and 155 cm^{-1} are due to the concerted vibrational motions of binuclear Bi–O entities, and the bands >155 cm^{-1} are ascribed to the vibrational modes of mononuclear Bi–O entities.[7] BO NS displays three characteristic Raman peaks at 118, 305 and 462 cm^{-1}, while BOC NS shows a single characteristic Raman peak at 154 cm^{-1} (Figure S17), which are in accordance with those in literatures.[8-10] However, BO/BOC exhibits several Raman peaks located at 88, 115, 135, 147, 178, 205, 305 and 442 cm^{-1}, which are far different from those of BO NS and BOC NS.

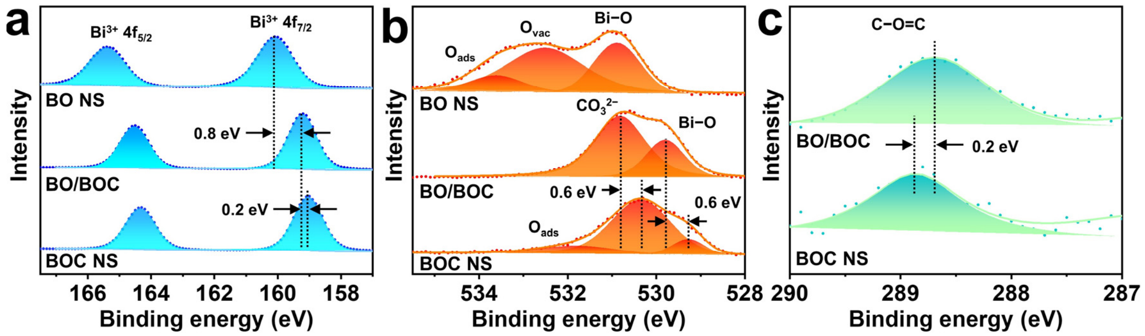

Figure S18. XPS spectra for (c) Bi 4f, (d) O 1s, and (e) C 1s signals of BO/BOC, BO NS and BOC NS.

BO NS has three oxygen species including surface adsorbed oxygen (O_{ads}), oxygen vacancy (O_{vac}) and Bi−O bond, while BO/BOC and BOC NS have similar oxygen components including CO_3^{2-} and Bi−O bond with unchanged binding energies (Figure S18b). The presence of O_{vac} in BO NS can be attributed to its hydrothermal synthesis under reducing conditions,[11,12] whereas the air-calcination used for BO/BOC introduces oxidative annealing that avoids the appearance of oxygen vacancies.[13] Besides, BO/BOC and BOC NS also display similar C 1s signals with the peak assigned to C−O=C (Figure S18c). These results interpret a closer surface chemical states of BO/BOC to $Bi_2O_2CO_3$.

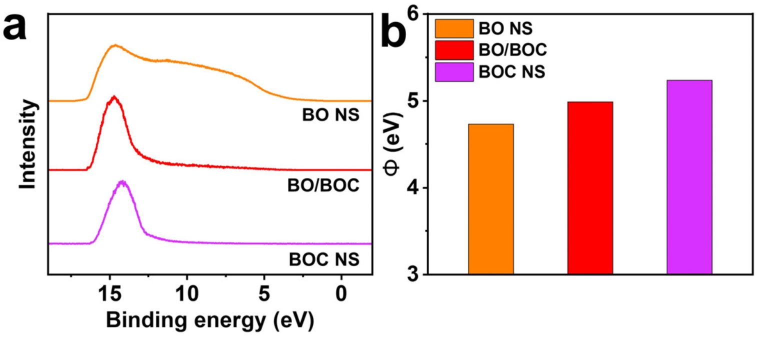

Figure S19. (a) UPS spectra and (b) work function values of BO/BOC, BO NS and BOC NS.

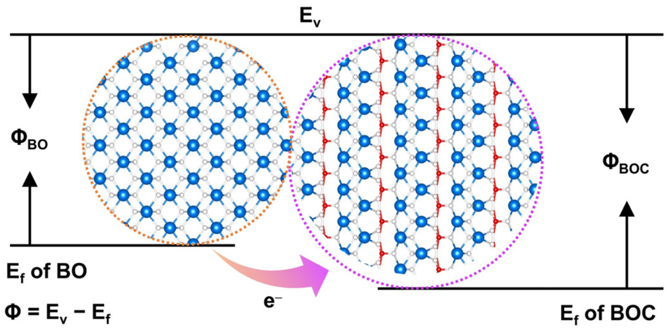

Figure S20. Schematic illustration of the directional electron transfer in BO/BOC.

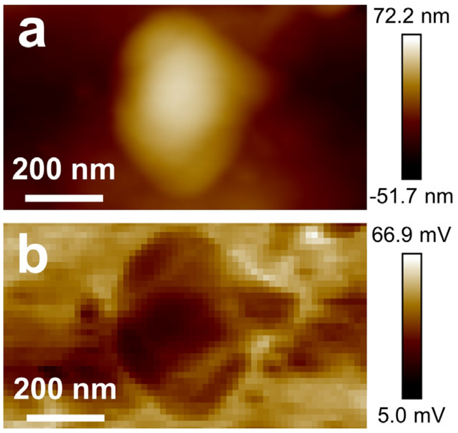

Figure S21. KPFM images showing the (a) topography and (b) surface potential distribution of BO/BOC.

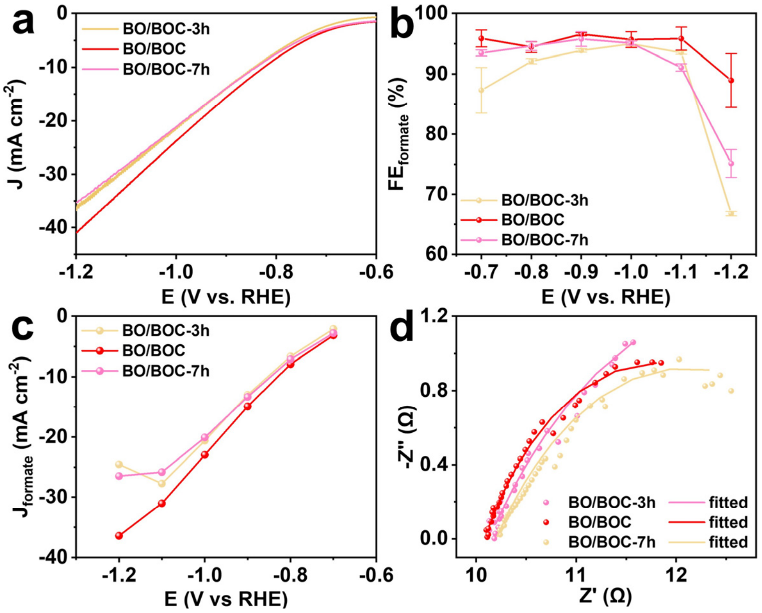

Figure S22. (a) LSV curves, (b) FE for formate, (c) $J_{formate}$ and (d) EIS tests of BO/BOC-3h, BO/BOC and BO/BOC-7h.

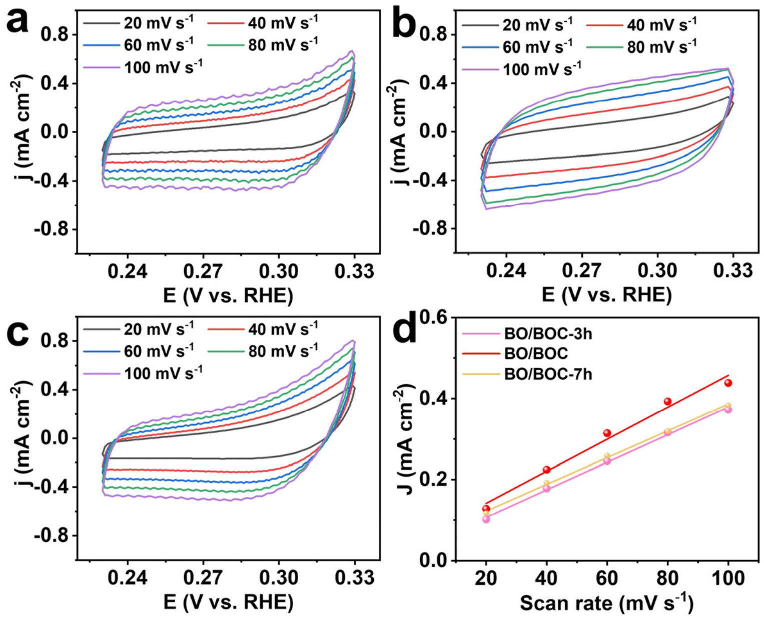

Figure S23. CV tests of (a) BO/BOC-3h, (b) BO/BOC and (c) BO/BOC-7h. (d) Comparison of capacitive current density versus scan rate of BO/BOC-3h, BO/BOC and BO/BOC-7h.

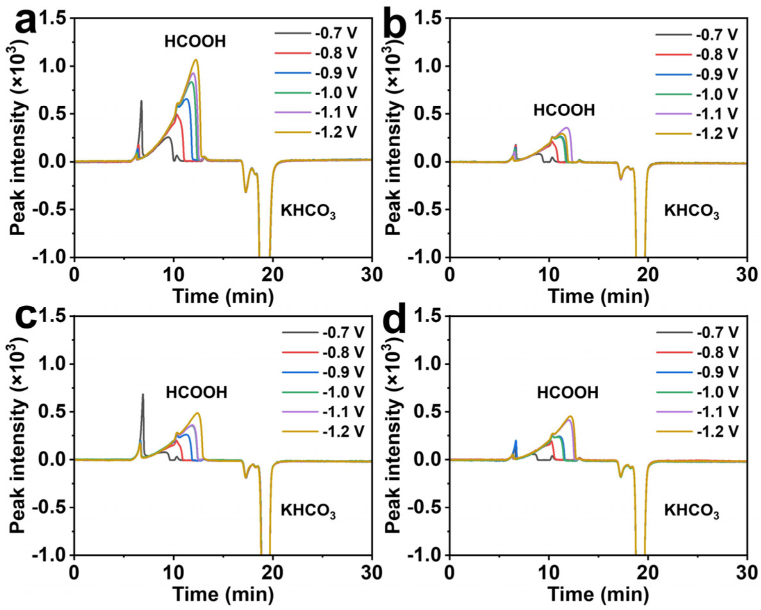

Figure S24. HPLC chromatograms for HCOOH production of (a) BO/BOC, (b) mixed BO/BOC NS, (c) BO NS and (d) BOC NS.

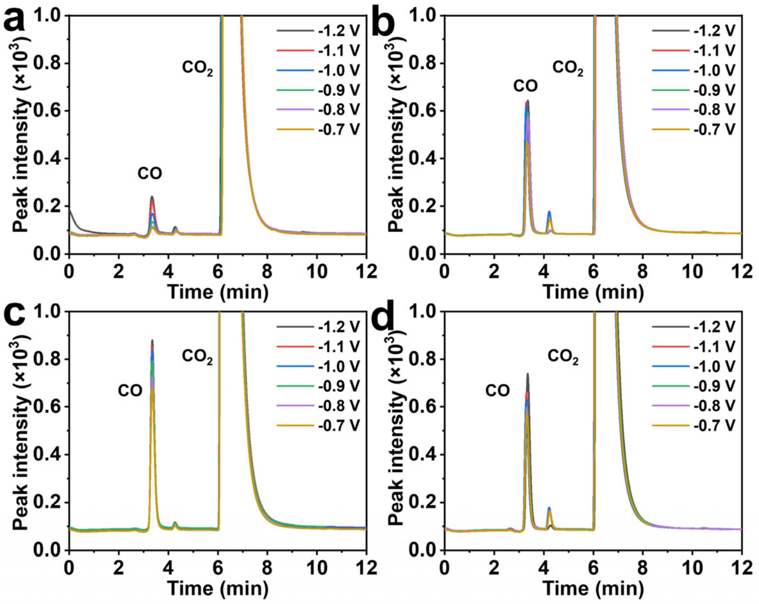

Figure S25. GC chromatograms for CO production of (a) BO/BOC, (b) mixed BO/BOC NS, (c) BO NS and (d) BOC NS.

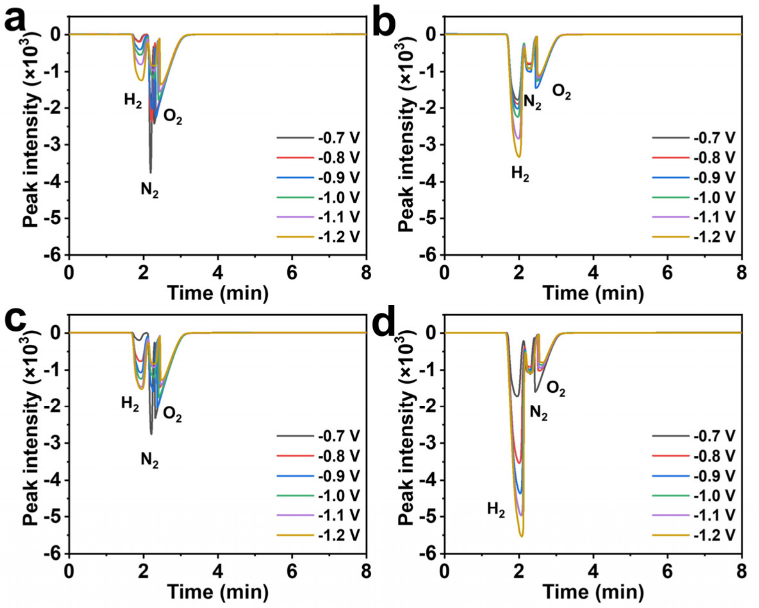

Figure S26. GC chromatograms for H_2 production of (a) BO/BOC, (b) mixed BO/BOC NS, (c) BO NS and (d) BOC NS.

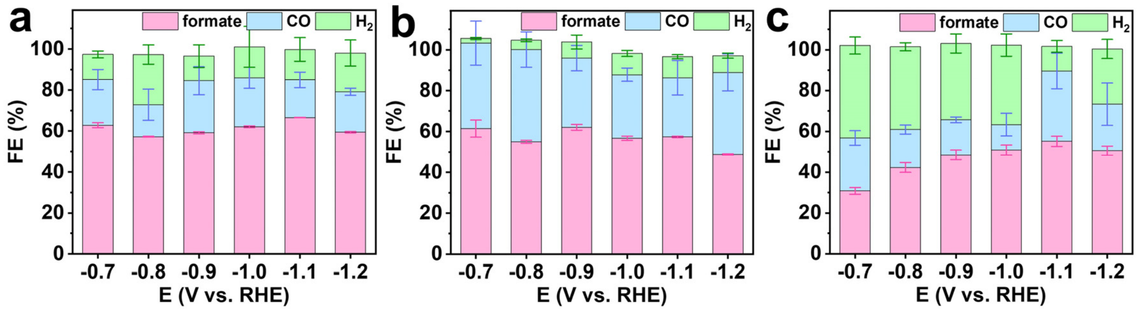

Figure S27. FEs of (a) mixed BO/BOC NS, (b) BO NS and (c) BOC NS. The data were averaged over three repeated measurements with the standard deviations marked by pink error bars for formate, light blue error bars for CO and light green error bars for H_2.

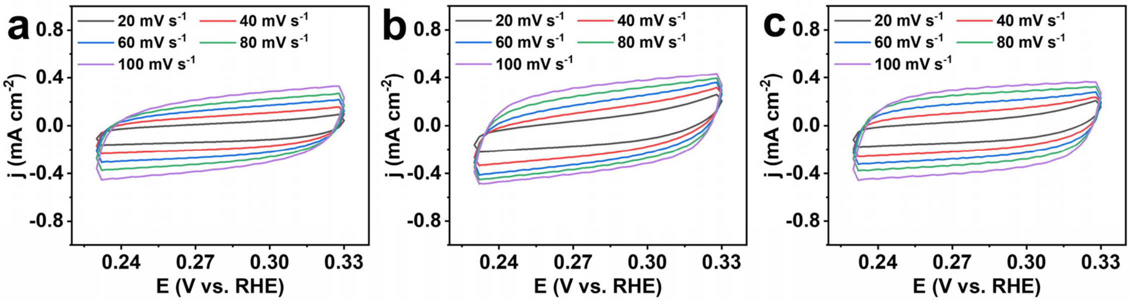

Figure S28. CV tests of (a) mixed BO/BOC NS, (b) BO NS and (c) BOC NS.

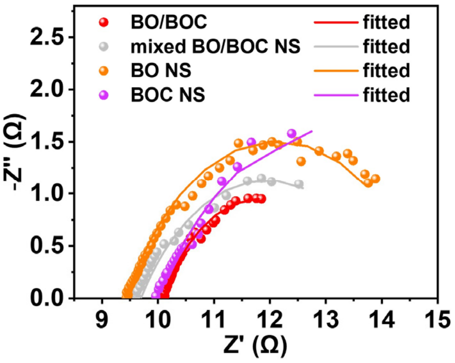

Figure S29. EIS tests and fitting results of BO/BOC, mixed BO/BOC NS, BO NS and BOC NS.

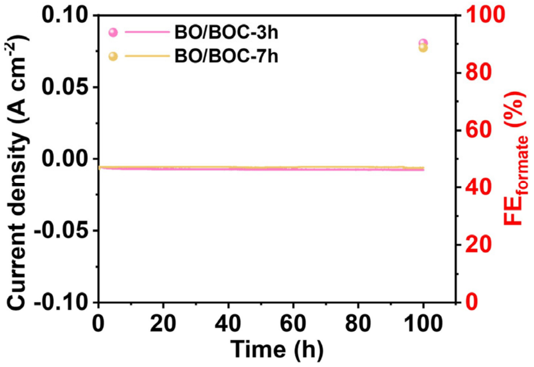

Figure S30. Stability test of BO/BOC-3 h and BO/BOC-7 h at −1.0 V in CO_2-saturated 0.5 M $KHCO_3$.

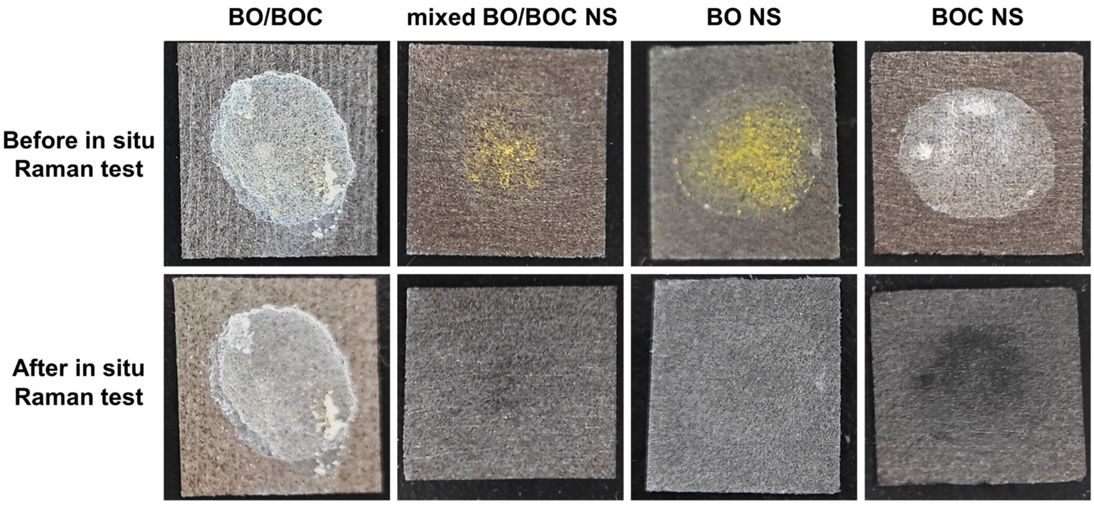

Figure S31. Optic photographs of BO/BOC, mixed BO/BOC NS, BO NS and BOC NS before and after in-situ Raman test.

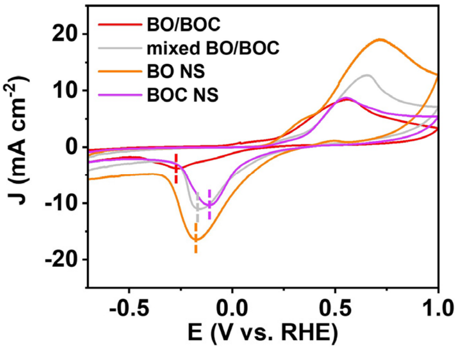

Figure S32. CV curves of BO/BOC, mixed BO/BOC, BO NS and BOC NS.

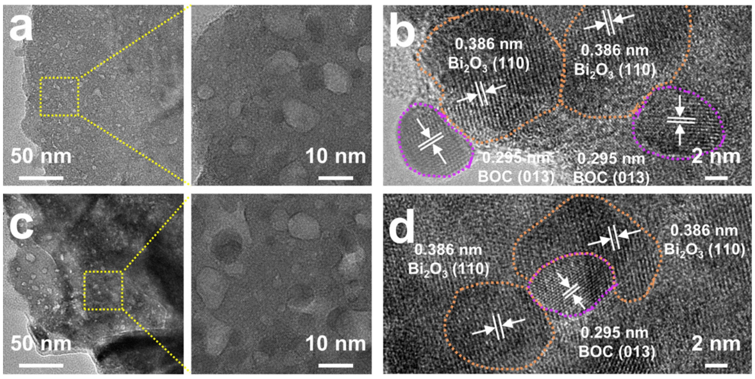

Figure S33. (a) TEM and (b) HRTEM images of BO/BOC after 48 h of durability test. (c) TEM and (d) HRTEM images of BO/BOC after 720 h of durability test.

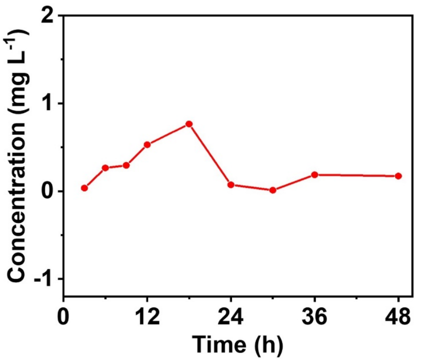

Figure S34. ICP-AES results of Bi content in electrolyte during the 48 h of durability test.

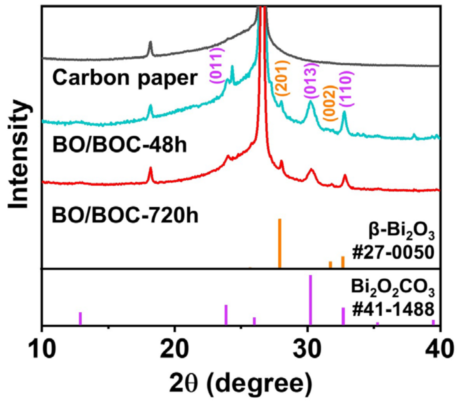

Figure S35. XRD pattern of BO/BOC after 48 h and 720 h of durability test.

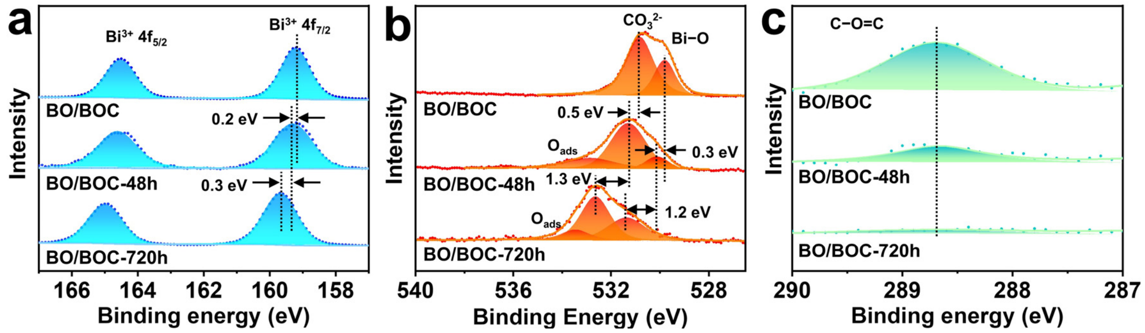

Figure S36. XPS spectra showing (f) Bi 4f, (g) O 1s, (h) C 1s signals of pristine BO/BOC and BO/BOC after 48 h and 720 h of durability test.

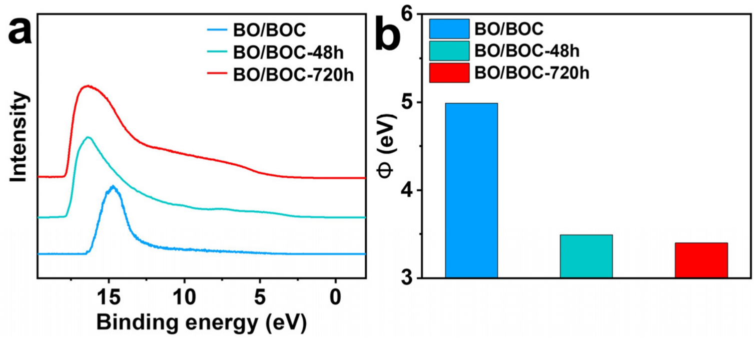

Figure S37. (a) UPS spectra and (b) work function values of pristine BO/BOC and BO/BOC after 48 h and 720 h of durability test.

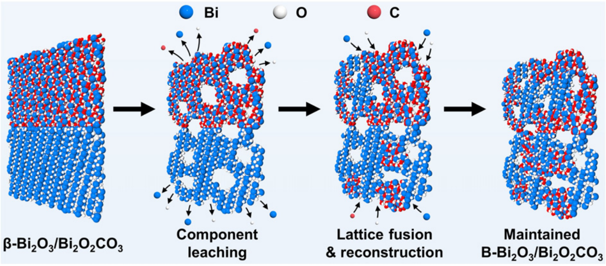

Figure S38. Schematic illustration of structural reconstruction mechanism of BO/BOC.

Table S1. EIS fitting results of BO/BOC, mixed BO/BOC NS, BO NS and BOC NS.

Catalysts	R_s (Ω)	R_{ct} (Ω)
BO/BOC	10.1	3.5
mixed BO/BOC NS	9.6	4.6
BO NS	9.4	5.4
BOC NS	10.0	7.0
BO/BOC-3 h	10.1	5.3
BO/BOC-7 h	10.2	3.8

R_S: Solution resistance

Table S2. Performance of BO/BOC in comparison to those of previously reported novel Bi-based and other metal-based catalysts toward eCO_2RR.

	**Catalyst**	**Electrolyte**	**FE_{max} (%)**	**J_{HCOOH} at FE_{max} (mA cm^{-2})**	**Durability time (h)**	**Reference**
1	BO/BOC	0.5 M $KHCO_3$	96.6	23.0	720	This work
2	Bi-NFs	0.1 M $KHCO_3$	92.3	11.4	16	Adv. Funct. Mater. 2023, 2301984
3	Bi_2O_3NSs@MCCM	0.1 M $KHCO_3$	93.8	~17	12	Angew. Chem. Int. Ed. 2019, 58, 13828-13833
4	BOC_R	0.5 M $KHCO_3$	95.7	N.A.	70	Small 2023, 2302878
5	Bi-Sn	0.1 M $KHCO_3$	93.9	9.3	10	Angew. Chem. Int. Ed. 2021, 60, 12554-12559
6	$Bi_{52}Sn_{46}In_2$	0.1 M $KHCO_3$	82	20.5	N.A.	Nano Lett. 2020, 20, 4403-4409
7	Bi-Sn alloy	0.1 M $KHCO_3$	78	~8	N.A.	Nat. Commun. 2019, 10, 4645
8	Bi-TiO_2-700	0.1 M $KHCO_3$	95.6	~7.5	70	J. Am. Chem. Soc. 2023, 145, 14133-14142
9	Bi@C NPs	0.5 M $KHCO_3$	95.0	10.5	18	Nano Lett. 2022, 22, 9107-9114
10	f-Bi_2O_3	0.1 M $KHCO_3$	87.0	15.5	N.A.	Adv. Funct. Mater. 2020, 30, 1906478
11	Bi_2S_3-PPy	0.5 M $KHCO_3$	91.2	~15	20	Energy Environ. Sci. 2023, 16, 3885-3898

12	BOC@GDY	0.5 M $NaHCO_3$	95.5	20.7	10	Sci. Bull. 2021, 66, 1533-1541
13	BOCNS	0.5 M $NaHCO_3$	85.0	N.A.	12	Angew. Chem. Int. Ed. 2018, 57, 13283-13287
14	Bi NS	0.1 M $KHCO_3$	92.0	~10	10	Angew. Chem. Int. Ed. 2021, 60, 18178-18184
15	Bi-ZMOF	0.1 M $KHCO_3$	91	~4	12	Angew. Chem. Int. Ed. 2023, 62, e202311223
16	S3-Cu_2O-70	0.1 M $KHCO_3$	88.7	5.7	80	Adv. Funct. Mater. 2023, 33, 2213145
17	Stanene	0.5 M $KHCO_3$	93.0	7	60	Adv. Energy Mater. 2024, 14, 2303889
18	R-In_2O_3	0.1 M $KHCO_3$	91.2	~13	80	ACS Catal. 2023, 13, 4021-4029
19	CPs@V11	0.5 M $KHCO_3$	90.1	6.87	20	Angew. Chem. Int. Ed. 2021, 60, 23394-23402
20	Sn-OH-5.9	0.1 M KCl	93.1	10.0	60	J. Am. Chem. Soc. 2019, 141, 2911-2915
21	NiSn-APC	0.5 M $KHCO_3$	86.1	20.8	N.A.	Angew. Chem. Int. Ed. 2021, 60, 7382-7388
22	VO-rich N-SnO_2 NS	0.1 M $KHCO_3$	83.0	6.7	10	Adv. Mater. 2021, 33, 2005113

N.A.: Not applicable

References

1. Hu, H.; Han, Q.; Liu, H.; Shen, Z.; Bi, H. Dual roles of basic bismuth nitrates in the composites: morphology regulation and heterojunction effects. *J. Mater. Sci.* **2020**, *55*, 11984–11998.
2. Jung, H. J.; Park, S.; Kim, K. D.; Kim, T. H.; Choi, M. Y.; Lee, K. Y. Fabrication of porous β-Bi_2O_3 nanoplates by phase transformation of bismuth precursor via low-temperature thermal decomposition process and their enhanced photocatalytic activity. *Colloid Surface A* **2018**, *550*, 37–45.
3. Huang, Y.; Wang, W.; Zhang, Q.; Cao, J.-j.; Huang, R.-j.; Ho, W.; Lee, S. C. In situ Fabrication of α-Bi_2O_3/$(BiO)_2CO_3$ Nanoplate Heterojunctions with Tunable Optical Property and Photocatalytic Activity. *Sci. Rep.* **2016**, *6*, 23435.
4. Karen, V. G.; Hernández-Gordillo, A.; Oros-Ruiz & Sandra, S.; Rodil, E. Microparticles of α-Bi2O3 Obtained from Bismuth Basic Nitrate [$Bi_6O_6(OH)_2(NO_3)_4·2H_2O$] with Photocatalytic Properties. *Top. Catal.* **2021**, *64*, 121–130.
5. Wang, X.; Mahbub, M. A. A.; Das, D.; Schuhmann, W. Design of Bismuth-Based Electrocatalysts for Carbon Dioxide Electroreduction. *ChemCatChem* **2024**, *16*(17), e202400601.
6. Tran-Phu, T.; Daiyan, R.; Fusco, Z.; Ma, Z.; Amal, R.; Tricoli, A. Nanostructured β-Bi_2O_3 Fractals on Carbon Fibers for Highly Selective CO_2 Electroreduction to Formate. *Adv. Funct. Mater.* **2020**, *30*(3), 1906478.
7. Dutta, A.; Zelocualtecatl Montiel, I. n.; Kiran, K.; Rieder, A.; Grozovski, V.; Gut, L.; Broekmann, P. A Tandem (Bi_2O_3 → Bi_{met}) Catalyst for Highly Efficient ec-CO_2 Conversion into Formate: Operando Raman Spectroscopic Evidence for a Reaction Pathway Change. *ACS Catal.* **2021**, *11* (9), 4988–5003.
8. Zhang, L.; Shi, Y.; Wang, Z.; Hu, C.; Shi, B.; Cao, X. Porous β-Bi_2O_3 with multiple vacancy associates on highly exposed active {220} facets for enhanced photocatalytic activity. *Appl. Catal. B-Environ.* **2020**, *265*, 118563.
9. Tang, S.-F.; Lu, X.-L.; Zhang, C.; Wei, Z.-W.; Si, R.; Lu, T.-B. Decorating graphdiyne on ultrathin bismuth subcarbonate nanosheets to promote CO_2 electroreduction to formate. *Sci. Bull.* **2021**, *66* (15), 1533–1541.
10. Liu, S.; Hu, B.; Zhao, J.; Jiang, W.; Feng, D.; Zhang, C.; Yao, W. Enhanced Electrocatalytic CO_2 Reduction of Bismuth Nanosheets with Introducing Surface Bismuth Subcarbonate. *Coatings* **2022**, *12* (2), 233.
11. Wu, Z.; Liao, T.; Wang, S.; Mudiyanselage, J. A.; Micallef, A. S.; Li, W.; O'Mullane, A. P.; Yang, J.; Luo, W.; Ostrikov, K.; et al. Conversion of Catalytically Inert 2D Bismuth Oxide Nanosheets for Effective Electrochemical Hydrogen Evolution Reaction Catalysis via Oxygen Vacancy Concentration Modulation. *Nano-Micro Lett.* **2022**, *14*, 90.
12. Yang, R.; Liang, B.; Zheng, S.; Hu, C.; Xu, Y.; Ma, Y.; Bai, Y.; Dai, K.; Tang, Y.; Zhang, C.; et al. Improving the Surface Oxygen Vacancy Concentration of Bi_2O_4 through the Pretreatment of the $NaBiO_3·2H_2O$ Precursor as a High-Performance Visible Light Photocatalyst. *Inorg. Chem.* **2022**, *61*(35), 14102–14114.
13. Qu, J.; Liu, W.; Liu, R.; He, J.; Liu, D.; Feng, Z.; Feng, Z.; Li, R.; Li, C. Evolution of oxygen vacancies in cerium dioxide at atomic scale under CO_2 reduction. *Chem. Catal.* **2023**, *3*(10), 100759.
